# Re: Effect of beam profile measurement on arc therapy plan quality assurance: A case study

**DOI:** 10.1002/acm2.12133

**Published:** 2017-07-14

**Authors:** Leonard H. Kim, Badal Juneja

**Affiliations:** ^1^ Department of Radiation Oncology MD Anderson Cancer Center at Cooper Camden NJ USA

## CONFLICT OF INTEREST

The authors declare no conflict of interest.

To the editor:

In our recent study on beam profile measurement and its effect on arc therapy plan quality assurance,^1^ we asked the question, but could not answer, why the reported effect was observed in only one of two described commissioning experiences, despite the use of similar equipment and methods. In the publication, we speculated the difference was due to the difference in machines being modeled (i.e., Varian TrueBeam vs. Elekta Infinity with Agility MLC.)

Since then, another experience commissioning a Varian TrueBeam suggests another possible answer. We observed that smoothing our measured data resulted in a noticeably shallower profile compared to unsmoothed measurements as well as Varian's “Representative Beam Data.” In essence, we had created a virtual “volume averaging” effect. This was unexpected, because we followed the same smoothing procedure described for the “Representative Beam Data” as well as that used by other institutions. Nevertheless, something about this particular combination of measurement equipment, acquisition technique, and post‐processing resulted in a noticeably altered profile. It is possible that similar circumstances resulted in the effects reported in our publication. It is thus advisable always to remember to assess the consistency of one's data at each step of the commissioning process.

## References

[1] Kim LH , Malajovich I , Reyhan ML , Xue J , Park JH . Effect of beam profile measurement on arc therapy plan quality assurance: a case study. J Appl Clin Med Phys. 2017;18:52–55. 28370918 10.1002/acm2.12071 PMC5689840

